# Optimization of a home hospitalization program for hematopoietic stem cell transplantation with ehealth integration and clinical pharmacist involvement

**DOI:** 10.3389/fimmu.2024.1397115

**Published:** 2024-06-11

**Authors:** Maria-Estela Moreno-Martinez, Mireia Riba, Irene García-Cadenas, Albert Esquirol, Marta Yusta, Sara Redondo, Anna De Dios, Jose Manuel Portos, Olga Aso, Angel Marcos-Fendian, Núria Font, Javier Briones, Rodrigo Martino, Anna Feliu

**Affiliations:** ^1^ Pharmacy Department, Hospital de la Santa Creu I Sant Pau, IIB Sant Pau, Barcelona, Spain; ^2^ School of Health Sciences Blanquerna, University Ramon Llull, Barcelona, Spain; ^3^ Hematology Department, Hospital de la Santa Creu i Sant Pau, IIB Sant Pau and José Carreras Leukemia Research Institutes, Universitat Autónoma de Barcelona, Barcelona, Spain; ^4^ Hematology Nursing Department, Hospital de la Santa Creu i Sant Pau, Barcelona, Spain; ^5^ Digital Health Department, Hospital de la Santa Creu i Sant Pau, Barcelona, Spain

**Keywords:** hospital-based home care, hematopoietic stem cell transplantation, chimeric antigen receptor T-cell, eHealth, clinical pharmacist, care model

## Abstract

Home hospitalization represents an alternative to traditional hospitalization, providing comparable clinical safety for hematological patients. At-home therapies can range from the delivery of intravenous antibiotics to more complex scenarios, such as the care during the early period after hematopoietic stem cell transplantation and chimeric antigen receptor T-cell therapy. Early discharge from conventional hospitalization is feasible and helps reduce hospital resources and waiting lists. The coordinated efforts of multidisciplinary teams, including hematologists, nurses, and pharmacists, ensure patient safety and continuity of care. The traditional model of home hospitalization relies on home visits and telephone consultations with physicians and nurses. However, the use of eHealth technologies, such as MY-Medula, can enhance communication and monitoring, and thereby improve patient outcomes with no additional costs. The active involvement of a clinical pharmacist in home hospitalization programs is essential, not only for the proper logistical management of the medication but also to ensure its appropriateness, optimize treatment, address queries from the team and patients, and promote adherence. In conclusion, the implementation of hematopoietic stem cell transplantation and chimeric antigen receptor T-cell therapy home hospitalization programs that use both an eHealth tool and a multidisciplinary care model can optimize patient care and improve quality of life without increasing healthcare costs.

## Introduction

Home hospitalization (HH) represents an alternative to traditional hospitalization and has demonstrated comparable clinical safety while offering advantages for both patients and healthcare systems. For patients, these benefits include a decreased risk of exposure to nosocomial infectious agents, as well as an increased perception of the quality of the overall process, with patients participating more in their own treatment as well as facilitating family reconciliation. For the healthcare systems, the benefits are reducing hospital stay and costs (1).

In Spain, the establishment of home-based care programs began in the early 1980s but their implementation is currently uneven, with differences in care models and resources. The HH services offered are diverse, ranging from the administration of parenteral drugs at home to specialized therapies such as post-hematopoietic stem cell transplantation (HSCT) care (2).

The first experiences in hematology hospitalization at home occurred in the late 1990s with home-based HSCT programs (3, 4). These programs allowed hospitals to reduce the resources used, to increase the number of therapies performed each year, and contributed to a shortening of waiting lists. Early discharge from the hospital after HSCT is a viable and feasible treatment strategy and is usually associated with a low incidence of complications and readmissions (5). Furthermore, HH has also been extended to other hematological therapies, including leukemia consolidation treatment. More recently, it has been applied to patients receiving chimeric antigen receptor T (CAR-T) cell therapy (6).

Regardless of whether the patient is at home or in the hospital the same standards of patient safety and care, as well as traceability and quality of the entire process must be ensured. HH patients must be followed by an experienced multidisciplinary team in HSCT, including hematologists, nurses, and clinical pharmacists (CP), among others. A well-articulated approach is necessary for medication management and adapting dispensing functions to address the unique needs of the care model. The inclusion of a CP in the HH team significantly contributes to improving patient safety and preventing medication-related problems (7–9).

Despite advances in home management, follow-up is usually based on home visits and telephone calls, with minimal support from new tools or technologies that would allow continuous communication between the patient and the care team (10). Likewise, the documentation of vital signs, a critical aspect in this population, is still carried out with paper-based methods that need subsequently to be added to the patient’s electronic medical record via computer.

The World Health Organization defines eHealth as the cost‐effective and safe use of information and communication technologies to support health and health‐related fields. eHealth includes different technologies including telehealth, telemedicine, mobile health (mHealth), electronic medical or health records, big data, wearables, and even artificial intelligence (11). The use of mHealth has the potential to enhance the flow of information between patients and healthcare professionals, facilitating early interventions and ensuring the safety and quality of care. Ultimately, this can improve health outcomes and patient experiences without incurring additional costs (12–15). Some of these technologies have already been developed to support the follow-up of HSCT patients with positive results (16, 17).

The objective of this article is to discuss the key points to be considered when designing an HH program that integrates eHealth into the interdisciplinary care process, whilst also focusing on the role of the CP.

## Design and optimization of an HH program

The design of an HH program requires the establishment of standard operating procedures (SOPs) with specific care pathways as well as a cohesive and committed multidisciplinary team, including experts in hematology, HSCT, or CAR-T cell therapy is critical for the program’s success. This team should be capable of anticipating both common and uncommon transplant-related complications, and each member must adapt roles to suit the requirements of the program. In our setting, we have worked to implement our HH program in 2024, and have learnt from other expert groups while, at the same time, adapting to our requirements. We planned daily phone calls from the hematologist to the patient, as well as home visits once-to-twice daily by the nurse, essential components of our program. Nurses, who are key members of the HH team, should report any concerns that arise during their visits. Meanwhile, the CP needs to continue with the normal activities recommended for a pharmacist involved in the care of HSCT and CAR-T cell therapy recipients (18–20), paying special attention to medication review to assess its appropriateness in the HH setting. This should include considering whether a ready-to-administer drug should be prepared, and adapting inpatient administration information to an at-home setting.

It is essential to establish clear criteria and procedures to identify patients eligible for an HH program, assessing the patient’s home and resources, and ensuring that the caregiver has the knowledge, skills, and attitudes to provide effective and efficient home care. Inclusion criteria at our center encompass patients preparing to undergo HSCT, CAR-T cell therapy, or intensive chemotherapy, without any comorbidities that could increase procedure-related morbidity and mortality. Additionally, patients must exhibit good performance status, live within a 60-minute radius of the hospital via public transport as well as having a home with adequate environmental hygiene, telephone access, and a 24-hour caregiver. Patients with a history of multi-drug resistant bacteria may not be considered for HH, although eligibility should be assessed on a case-by-case basis. Exclusion criteria include confirmed platelet transfusion refractoriness, severe uncontrolled psychiatric disease, residence in a nursing home or a language barrier. Eligible patients are identified at the initial pre-procedure visit and, if accepted, an HH team member conducts a first visit to confirm practical issues such as the adequacy of home facilities.

The role of the caregiver is fundamental in an HH program, as they are responsible for constant supervision, and assistance with hygiene, feeding, and medication. Additionally, the caregiver must acquire sufficient knowledge and understanding of the patient’s specific needs and maintain communication with the healthcare team in the event of complications. Adequate cultural competence of the patient and the primary caregiver, along with an understanding of the HH program and coordination with the care team, is essential. Caregivers are trained, along with patients, on how to identify significant adverse events and instructed to directly contact the HH team in the event of such an occurrence.

The administration of the conditioning regimen and the infusion of hematopoietic stem cells or CAR-T cell therapy are conducted in the hematology ward. It is essential that there is fluent communication between the home and conventional inpatient team to judge whether the patient is ready for a home transfer. In the case of HSCT, this will be the day after the infusion, while with CAR-T cell therapy, this moment can vary depending on the drug administered, usually ranging from 3 to 7 days after infusion. Conversely, in the event of complications requiring urgent care or hospitalization, the inpatient team should be notified, and an inpatient bed should be available in the hematology ward to avoid unnecessary visits to the emergency room.

Some of the SOPs recommended to be adopted for the HH program include the management of the most common toxicities such as gastrointestinal disorders, mucositis, neutropenia, and the prevention of infections. Broad-spectrum antimicrobials with good oral tolerability are recommended, covering the most common infections in HSCT or CAR-T cell recipients, for example, in the case of allo-HSCT, quinolones, fluconazole and acyclovir are usually prescribed as antimicrobial prophylaxis (6).

It is also important to document the criteria for patient discharge from the HH program, which in our center include the resolution of all extrahematologic toxicities, a neutrophil count above 0.5 x 10^9^/l, absence of hemorrhagic diathesis, and a stable platelet count above 10 x 10^9^/l. Furthermore, the patient must not need blood product transfusion more than twice a week, demonstrate the absence of potentially infectious fever, significant vomiting or diarrhea, and exhibit acceptable food and fluid intake. Additionally, the patient must demonstrate an understanding of the post-discharge medications, care instructions, and lifestyle changes.

## Use of technologies in the HH program

The use of information and communication technologies to enhance collaboration among all the members of the multidisciplinary team involved in the HH program, as well as with the patient and/or caregiver, is strongly recommended whenever feasible (21).

One technology that supports the HH program involves the creation of virtual beds for HH patients within the Computerized Prescribed Order Entry (CPOE) system, which ensures that medication management aligns with traditional inpatient settings protocols. Upon transitioning the patient to the HH setting, the medical team conducts a comprehensive review of the patient’s active medications, and the CP verifies the treatment plan before dispensing the prescribed medications. The multidisciplinary team provides appropriate education and written information about the treatment plan to the patient and caregivers. The HH physician is responsible for reviewing and adjusting the pharmacologic regimen in the CPOE in response to any new situation. The CP should validate and dispense the medication to the HH nurse who, in turn, delivers it when going to the patient’s home.

A portable device such as a tablet or a laptop with remote access to the hospital system is essential for nurses since this allows them to access all patient information including clinical history, medications, blood tests and vital signs. Nurses can record vital signs, register medication administration, and update the clinical history. A pre-printed sheet can be provided, or an eHealth solution implemented, for self-administered medication by the patient or caregiver, and for documenting symptoms or adverse events. If the eHealth solution is available, it will allow the integration of all records generated by the patient or caregiver in the electronic clinical history.

**Figure 1:**
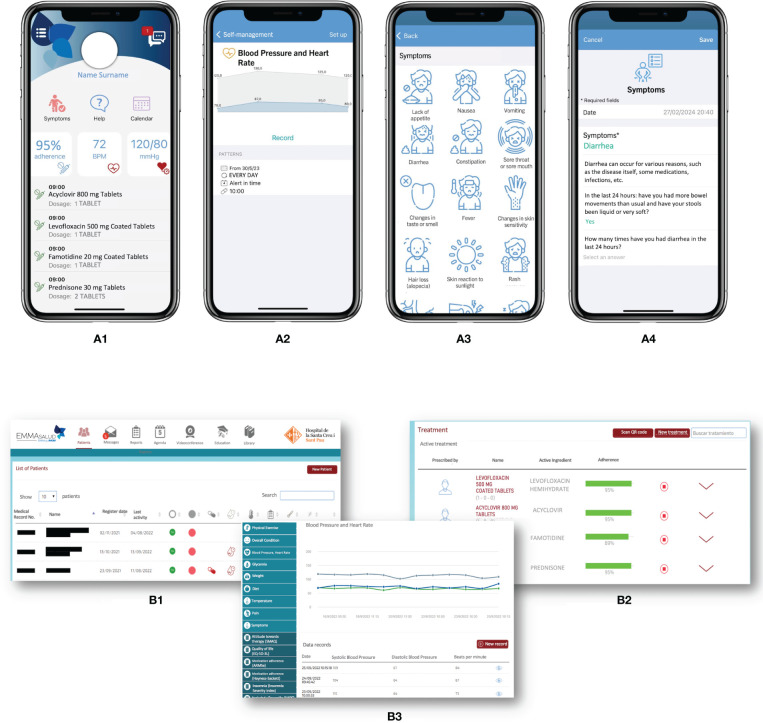
MY-Medula mobile phone app, version for patients and for healthcare professionals (16). **(A)** Different modules of the mobile phone app for patients are displayed: (1) home page with direct access to, symptoms, treatment, and agenda modules with daily tasks; (2) graphic of blood pressure and heart rate reported; (3) symptom reporting list; and (4) diarrhea symptom report. **(B)** Different modules of the web page for healthcare professionals are displayed: (1) home page with access to all patients and a vital signs monitoring system based on color code alerts; (2) treatment screen on which the healthcare professional can access the patient’s medication list and monitor adherence; (3) access to the patient’s self-records in graphical and data table formats. Adapted from Redondo S, et al. (16).

In 2013, our Pharmacy department led the way in implementating mHealth programs specifically designed for complex chronic populations, including heart transplant patients (mHeart) and complex chronic patients (Medplan+, renamed MyPlan) (22–24). Based on the initial outcomes of these pilot initiatives, we proposed the development of the EMMASalud-MY-Medula application (App), designed for HSCT outpatient follow-up, which is also suitable for patients enrolled in an HH program. The mobile App enables patients or caregivers to self-monitor and record a range of vital signs (blood pressure, temperature, heart rate, weight or glycemia), medication and food intake, mood, symptoms, or adverse effects following the Common Terminology Criteria for Adverse Events (CTCAE) v5 standard (diarrhea, constipation, insomnia, vomiting, nausea, asthenia, headache, pain, skin problems, and others) (**Figure 1A**). The App allows users to visualize and customize daily tasks, receive medication reminders, and features a real-time bidirectional messaging service for prompt communication with healthcare professionals. It also includes an educational section with updated information on the most commonly prescribed drugs, post-HSCT complications and diet and exercise recommendations (16).

An associated website allows healthcare professionals to review patients’ symptoms and adverse events, treatment adherence, and other health registers (**Figure 1B**). MY-Medula synchronizes with the hospital’s electronic medical records and allows importing patients’ sociodemographic data into the App. Patient reported outcome measures (PROMs) are added to the hospital medical record weekly and can also be downloaded from the App whenever needed. **Table 1** provides a detailed overview of the functionalities of the patient’ and healthcare modules and their clinical applications.

**Table 1:** My-Medula patient and professional profile modules, components, and clinical use.

Patient Modules	Components and clinical use
**Treatment**	Drug catalog synchronized with the Spanish National Formulary Medication list includes active treatment list and inactive drugs New medication list included by patient or caregiver. Pharmacist validates the treatment, checking for interactions All medication included in the active treatment list generates a visual and sonorous small alarm to help the patient remember taking their medication. Non pharmacologic treatments can also be included
**Records**	Vital signs (i.e., blood pressure, heart-rate, temperature, pain), biomeasurements (i.e., weight, height, blood glucose), dietary intake, exercise data Health questionnaires: i.e., medication adherence (ARMSe), anxiety and depression (HADS) Symptoms or adverse effects generate an alert to clinicians. A color code (red-orange-green) of the alert helps to prioritize intervention
**Communication**	A real-time bidirectional chat between patient and healthcare professional allows the attachment of images, files and audio recordings.
**Agenda**	A text alert can be triggered on the patient’s cell phone for drug intake alert and test appointment reminders.
**Information**	Information on healthy lifestyles (multimedia files, links to videos), on treatment and useful information about the team or the health center.
**Help**	A help center service to solve both technical and functional problems Clinical contact data The legal requirements accepted at first use are always available for consultation.
Healthcare Modules	Component and clinical use
**Patient Reports**	On the main page there is a list of active patients with colored traffic lights and registration alerts to help the clinicians prioritize their intervention. They can review and track: - New treatments introduced pending pharmacist validation - Vital signs - Symptoms and adverse events - Completion of questionnaires - Pending messages - Patient adherence - Patient inactivity (by filtering by the last activity date) All patient reported experience measures (PREMs) and patient reported outcomes measures (PROMs) recorded by patients can be downloaded, and filtered by date in Excel format.
**Treatment Prescription**	Pharmacological treatment is usually prescribed by pharmacist, patient or caregiver from a drop-down list of drugs updated from the Spanish National Formulary. Pharmacist validates the treatment, that has to be similar to computer order prescription. Tailored recommendations can be added

ARMSe, Adherence to Refill and Medication Scale; HADS, Hospital Anxiety and Depression Scale. Adapted from Redondo S, et al. (16).

The process of registering and activating the drugs in the App, with their respective dosages is performed by the CP after validating the updated treatment in the CPOE program. Consequently, the treatment information that the patient can see on the App is similar to the information they would find on the printed prescription chart from the CPOE program. Future integration to avoid medication transcription is being considered for subsequent technology improvements.

## Discussion

Typically, HH programs are designed to optimize resource utilization, reduce hospital stay, prevent nosocomial infections, and improve the quality of the entire process. The initial reports proving the viability of HH programs in hematological patients focused primarily on autologous HSCT, particularly in patients diagnosed with multiple myeloma (3, 4). Over time, these home or outpatient programs have expanded to include allo-HSCT, encompassing patients receiving post-transplant cyclophosphamide (5, 25–28), as well as more recently CAR-T therapies (6, 29).

One of the main challenges when initiating an HH program is to determine patient inclusion criteria and identify each candidate’s eligibility, as defined and assessed by the healthcare team. Two key points influencing eligibility are the distance from the patient’s home to the hospital and the 24-hour availability of a caregiver. While some authors have proposed a 30- or 60- minute driving radius as a requisite for inclusion in the HH program (6, 25), our approach extends eligibility to homes no more than 60 minutes away from the hospital by public transportation, irrespective of whether they have a car and a driver available full time. Immediate family members are usually the main caregivers, assisting with daily tasks and other issues such as treatment management. The availability of a full-time caregiver is a critical requirement for inclusion in an HH program since this support can contribute to optimal clinical outcomes for HSCT patients. Indeed, studies have demonstrated that HSCT patients who perceive themselves to have good social support tend to report a better quality of life and less psychological distress (30).

Coordination between all members of the healthcare team is essential for the success of an HH program, including physicians, nurses, and pharmacists. Home care visits by nurses have proved to be an effective strategy in reducing the need for hospitalization among cancer patients (31). Between 14 and 30 days is the typical hospital stay for an autologous or allogeneic HSCT or CAR-T therapy and this can be reduced to less than seven days with HH, thereby reducing treatment cost (1, 25, 27, 31). However, the total home stay may be longer, depending on the characteristics of the patients enrolled in the study (32). Potential clinical benefits include a decreased risk of nosocomial infections, febrile neutropenia, graft-versus-host disease and transplant-related mortality (26, 27, 33). A gradual approach, with one or two patients at a time and frequent meetings with the entire team, provides an optimal setting to implement adjustments to procedures and evaluate the program. A comprehensive evaluation will be conducted at the end of the first year after the implementation of the HH program to assess the outcomes and costs associated with this initiative.

A key factor for the success of these programs is effective communication. CPOE programs and other applications can facilitate communication by using the same prescription and follow-up program for both hospitalized and home-based patients. This ensures that all healthcare team members have access to information, in this way facilitating a smooth transition between different levels of care in alignment with Joint Accreditation Committee ISCT-Europe & EBMT (JACIE) recommendations. The CP plays an essential role by assessing the appropriateness and optimization of treatments, evaluating medication adherence, drug-drug or drug-food interactions, and assuring the safe management of drugs in the patient’s home. For this, the CP needs to be able to effectively clarify any doubts raised by physicians, nurses, patients, and/or caregivers. Integrating a CP into an HH team has been shown to reduce drug-related problems and save costs (9, 34).

The use of eHealth is perceived by some authors as an opportunity to improve care in the HH setting, despite its limited implementation. A small number of mobile Apps have been developed in the field of hematology (35, 36), including those designed to enhance disease management, such as improving adherence to treatment and maintaining patient’s motivation to engage in healthy behaviors. In a qualitative study by Amonoo et al., HSCT patients described the need for more guidance and resources to understand the HSCT process, not only for themselves but also for caregivers, and in this respect mobile Apps can be very helpful (37). There is a lack of information about the psychological impact of these therapies and the HH program on patients and caregivers, and eHealth can help us identify situations that require our intervention.

Our center has experience in enrolling heart transplant recipients (mHeart) and chronic complex patients (MyPlan) in mHealth programs, and the results have been high levels of acceptance, usability, and improved adherence to treatment (22–24). HSCT patients usually have good treatment adherence although they do not always take drugs as prescribed (38). The pilot study of MY-Medula, which employed the same technology but adapted for hematological patients, helped improve communication between patients and the HSCT healthcare team, enabling the early detection of complications such as certain psychological disorders and the implementation of interventions based on PROMs. Furthermore, patients also demonstrated a high level of satisfaction using the technology (16). The patient information module of MY-Medula provides patients and caregivers with multimedia files and links to videos containing information selected by the multidisciplinary team. This information is designed to enhance patients’ and caregivers’ knowledge about their condition and complications, improve their adherence to the technology and reduce their concerns.

The CP has been acknowledged as an essential member of the multidisciplinary team in several settings, including the pharmaceutical care of HSCT and CAR-T cell patients. Some of the pharmacist’s core competencies include medication management and counseling, symptom management, therapeutic drug monitoring, discharge planning and transitions of care, and patient and caregiver education. All of these are fully applicable to HH programs (18, 19). The CP can offer assistance by reviewing treatment, facilitating medication access and ensuring proper administration instructions in case of HH (7, 8). We have observed that the communication between the patient and the CP has increased through mHealth, and that non-urgent consultations have been resolved with pharmaceutical care becoming easier (39). We believe that this technology can be integrated into the HH setting. The pharmacist is the one who registers and activates the medication in the App, thereby facilitating the traceability of the entire process and assuring proper drug delivery, such as whether the medication should be taken on an empty stomach or with food.

In conclusion, the integration of a CP in HH multidisciplinary teams and the incorporation of eHealth to HH programs have the potential to enhance patient monitoring, medication management, patient and caregiver education, and to improve quality of life, and reduce costs.

## Data availability statement

The original contributions presented in the study are included in the article/supplementary material. Further inquiries can be directed to the corresponding author.

## Ethics statement

Written informed consent was obtained from the individual for the publication of any potentially identifiable images or data included in this article.

## Author contributions

MM: Writing – review & editing, Writing – original draft, Conceptualization. MR: Writing – review & editing, Conceptualization. IG: Writing – review & editing, Validation, Conceptualization. AE: Writing – review & editing, Validation. MY: Writing – review & editing. SR: Writing – review & editing. AD: Writing – review & editing. JP: Writing – review & editing. OA: Writing – review & editing. AM: Writing – review & editing. NF: Writing – review & editing. JB: Writing – review & editing. RM: Writing – review & editing, Conceptualization. AF: Writing – review & editing, Conceptualization.
